# Physical Activity and the Improvement of Autonomy, Functional Ability, Subjective Health, and Social Relationships in Women over the Age of 60

**DOI:** 10.3390/ijerph18136926

**Published:** 2021-06-28

**Authors:** María Antonia Parra-Rizo, Gema Sanchís-Soler

**Affiliations:** 1 Department of Health Psychology, Faculty of Social and Health Sciences, Campus of Elche, Miguel Hernandez University (UMH), 03202 Elche, Spain; 2 Department of General Didactics and Specific Didactics, University of Alicante, 03690 Alicante, Spain; gema.sanchis@ua.es

**Keywords:** physical activity, functional ability, functional autonomy, elderly, health, quality of life

## Abstract

Regarding functional ability, autonomy, promotion of social relationships and health, little scientific evidence has been found of physical practice in active women over 60 years of age. Hence, the goal of this study was to assess the functional abilities and autonomy, social relationships and subjective health of physically active older women according to the level of activity practiced. The IPAQ and CUBRECAVI scales were applied to a sample of 257 women between 61 and 93 years old (M = 69.44, SD = 4.61). Statistically significant outcomes were obtained in functional ability and autonomy according to their level of physical activity (*p* = 0.001): greater satisfaction and frequency of social relationships with a mild level of physical activity (*p* = 0.011), and statistically significant differences in the degree of satisfaction with their health according to the level of physical activity they practice (*p* < 0.001). The results showed that those with high physical activity obtained better levels of functional abilities and autonomy. Additionally, dissatisfaction with one’s own health is associated with low levels of physical activity. In conclusion, it could be said that the practice of mild physical exercise in older women encourages greater autonomy and functional ability for activities of daily living, which results in independence in everyday life in addition to fostering social links as well as gaining a better satisfaction with their own health, with the socio-emotional benefits that this can bring.

## 1. Introduction

In the near future, approximately in 2050, 22% of the international population will be over 65 years of age (2.1 million people) [1,2], and considering life expectancy, the majority of these people will be women. For this reason, various studies suggest the need to identify those aspects that can contribute to a successful and happy aging, namely: improving quality of life, and understanding that the set of personal, socio-environmental, subjective and objective factors such as leisure activities, social interactions, health, and social support, among others such as functional ability and autonomy are those necessary to take care of oneself, constituting a critical factor for health and well-being that promote positive and healthy aging and reduce the risk factors that cause different diseases and disabilities at this stage of life in general, and for women in particular [3].

In this regard, the regular practice of physical activity allows maintaining and enhancing physical, physiological and psychological health, favouring functional independence as well as the absence of diseases in old age—in fact, physical activity as any body movement made by skeletal muscles, with the consequent consumption of energy, all movement, even during leisure time, to move to and from certain places, or as part of person’s work. Common physical activities include walking, cycling, playing sports, and participating in recreational activities and games [4,5]. In fact, some authors point out that a healthy lifestyle also has an impact on good mental health as it enhances positive emotions such as well-being, happiness and optimism [3].

As has been said above, one of the ways to enhance these variables is by the practice of physical activity. On the contrary, and as various research concluded, a sedentary lifestyle negatively affects health and consequently quality of life. This was the conclusion reached in a study conducted with a sample of 4276 older people [6]. In the same vein, 2 cross-sectional studies carried out with samples of 3201 and 3543 older people, respectively, showed that those who spent more time without practicing physical activity (more than 8 h a day in a sedentary state) had a poorer psychological, physical and social quality of life [7]. In fact, the regular practice of physical activity is related to fewer chronic health disorders and depressive symptoms [8]. In the same way, in their quasi-experimental randomized controlled trial conducted with 54 older women, significant interactions were found between physical activity and improved physical strength [9,10].

Thus, in recent research that used a sample of 139 elderly people (71.94% women), it was observed that the group that practiced physical activity on a regular basis had better functional capacity as well as a greater degree of independence [11]. Another study carried out with a sample made up of 152 women over 60 years of age corroborated the relationship between the practice of physical activity and better health, functional capacity and independence, confirming the increased risk of worsening in these variables when it came to inactive women, regardless of the health variables [12].

Similarly, a study that used a sample of 200 widows over 60 years old concluded that participation in recreational activities not only helped to mitigate the negative effects of loneliness, but enhanced life skills, as well as mental and social well-being [13]. In this sense, there is evidence that physical activity protects against depression [14,15], in addition to producing improvements in psychological well-being and quality of life related to health, although it has a greater impact among individuals who practice team sports [16]. Along these lines, after analyzing 34 older women out of a total sample made up of 39 individuals, an investigation concluded that physical activity helped manage emotions and improved socialization [17]. This benefit is bidirectional, that is, contact with friends favoured the practice of physical activity and this, in turn, favoured cognitive capacity and function [18].

From this interaction between physical activity and the improvement in social relationships, psychological and functional status and independence, an improvement in subjective perception of health is also obtained [5], which entails a decrease in the degree of dependence [19], as well as a perception of greater body control and self-esteem [20]. In this regard, it should be noted that, as with other variables, there could be a relationship concerning the intensity of physical activity practiced and the subjective perception of health; the higher the intensity, the better the subjective perception of wellbeing is, and vice versa [21].

Therefore, it can be concluded that health, social relationships, functional autonomy and being active are fundamental factors in order to achieve a good quality of life in older women [22].

However, despite the scientific literature discussed above, little research has paid attention to the relationship between the variables presented in this work (quality of life, level of physical activity, autonomy, subjective health, social relationships, and functional abilities) in physically active older women. And their study would allow promoting health as well as specific interventions applied to older women. Therefore, starting from a sample of physically active elderly women, the general goal of this work is to study whether women who practice a significant amount of physical exercise have the best standards of personal quality-of-life factors compared to those who perform mild or low levels of physical exercise. In particular, the aim was to determine the following aspects:

(a) If there are differences in functional abilities and autonomy between older women with a high level of physical activity versus those with a mild or low level;
(b) If there are differences in social relationships between older women with a high level of physical activity compared to those with a mild or low level; and
(c) If there are differences in satisfaction with health between older women with a high level of physical activity compared to those with a mild or low level.

## 2. Materials and Methods

### 2.1. Participants

A cross-sectional study was conducted with a sample of 257 older woman residing in Alicante (Spain). When it comes to age, the minimum was 61 years and the maximum 93, the average age being 69.44 years (SD = 4.61). Their marital status was 56.4% married, 23.3% widows, 8.2% single, 7.8% divorced and 4.3% other than the previous ones; 41.2% of these women lived alone.

The inclusion criteria were: (1) older than 60 years; (2) physically active; and (3) physically active for more than a year. These data were obtained through the answer to a question about how long they had been doing physical activity on a regular basis. And the exclusion criteria were: older women who cannot read the questionnaire.

In terms of health, the use of tobacco and alcohol as well as the existence of physical and psychological illnesses were analysed. In terms of habits, 93% were nonsmokers and 59.5% of them never used alcohol, while 4.3% smoked more than five cigarettes/day and 19.1% drank alcohol daily. Regarding their illnesses, 70.4% of them did not have any physical ailment and 85.6% did not have any psychological illness.

### 2.2. Instruments

#### 2.2.1. International Physical Activity Questionnaire, IPAQ

The Physical Activity Questionnaire [23] assesses three categories of exercises according to their intensity: low, mild and high, and classifies the members of this study into three activity levels: intense (<3 METs), mild (3–6 METs) and low (>6 METs) [24]. It has seven items that transform the minutes, hours and days of physical practice, and the questionnaire gives their categories of low (>6 METs), medium (3–6 METs) and high intensity (<3 METs). Individuals with high activity levels exercise daily at a minimum 1 h more of a mild intensity activity over the basal activity level, or half an hour of a high intensity activity above the basal daily level. Individuals with mild activity levels engage in at least half an hour more of physical activity of mild intensity almost every day. Individuals with low activity levels are those who do not accomplish mild or intense activity levels. A short version requested that the participants respond to seven items connected to the physical activity that they had performed in the past week. We found questions such as “how much time in total did you spend in vigorous physical activity on one of those days?”; “how much total time did you spend in moderate physical activity on one of those days?” The questionnaire has a reliability coefficient of 0.65 (rs = 0.76; 95% CI: 0.73–0.77) and was recently used in studies related to older adults [25,26].

#### 2.2.2. Brief Questionnaire on Quality of Life

This questionnaire consists of 21 subscales grouped into nine scales, among which subjective health and functional skills (functional autonomy and activities of daily living) were considered in order to carry out this study. This questionnaire is highly recommended to assess quality of life [27]. Participants must value the degree of satisfaction or the frequency of the items. We found questions such as “Are you satisfied with your state of health?” on a Likert response scale from 0 “nothing” to 3 “a lot of”. The internal consistency of the scales varies between 0.70 and 0.92. This questionnaire was applied recently to evaluate the standard of living in elderly people [28].

### 2.3. Procedure

Individuals responsible for managing 38 sports activities and cultural facilities in Alicante were reached so they could provide information on the topic. An informed consent form together with the questionnaire that had to be completed, as well as an explanation of the research and its objectives, were given to each of the 18 sports and cultural facilities that consented to collaborate.

In order to guarantee the confidentiality of the data, questionnaires were distributed in a packet that, once completed, had to be sent back in a follow-up meeting that was set up in that moment.

### 2.4. Analysis of Data

With the purpose of studying the differences among the participants according to their level of physical activity, univariate analysis of variance (ANOVAs) and multivariate analyses (MANOVAs) were applied to the quantitative variables, and the chi-square test was applied to the qualitative variables.

In order to estimate the effect size, ƞ2 was used in the ANOVAs and MANOVAs and, in the qualitative variables, the Phi Coefficient and Cramer’s V were considered (dependent on the magnitude of the contingency tables).

The established significance value is < 0.05.

The data analyses were completed with the SPSS statistical package, version 23.0. (IBM corp., Armonk, NY, USA).

## 3. Results

### 3.1. Subjective Health

The analysis of the scores that evaluate the satisfaction of the participants with their health status (subjective health) (Table 1) showed that there are statistically significant differences in the degree of satisfaction they have according to the level of physical activity they practice (χ^2^ (6, *n* = 257) = 25.96; *p* < 0.001) with a little connection between variables (VCramer = 0.225). The degrees of satisfaction in which the individuals differ according to their activity was analysed, and the results indicate differences between the degree of those who do not have any satisfaction with the other degrees (χ2(2, *n* = 257) = 8.15; *p* = 0.017) with a little connection between the variables (VCramer = 0.178) being the differences between those of intense activity with those of mild/low activity (3.8% vs. 11.8%) (χ2 (1, *n* = 257) = 4.96; *p* = 0.026; Phi = −0.139) and between those of low activity and those of intense/mild activity (18.9% vs. 6.8%) (χ2 (1, *n* = 257) = 5.93; *p* = 0.015; Phi = 0.152); between the grade of those who are quite satisfied and the other grades (χ2 (2, *n* = 257) = 21.13; *p* < 0.001) with a little connection between the variables (VCramer = 0.287) being the differences between intense activity and mild/low activity (66.4% vs. 39.2%) (χ2 (1, *n* = 257) = 18.23; *p* < 0.001; Phi = 0.266), among those of mild activity with those of intense/low activity (43.1% versus 56.0%) (χ2 (1, *n* = 257) = 4.25; *p* = 0.039; Phi = −0.129) and between those with low activity and those with intense/mild activity (27.0% versus 54.1%) (χ2 (1, *n* = 257) = 9.28; *p* = 0.002; Phi = −0.190); and among those in which the degree of satisfaction they have is high with the other degrees (χ2 (2, *n* = 257) = 7.72; *p* = 0.021) with a little connection between the variables (VCramer = 0.173) being the differences between those of high activity and those of mild/low activity (10.6% vs. 22.2%) (χ2 (1, *n* = 257) = 5.81; *p* = 0.016; Phi = −0.150) and among those of low activity with those of high/mild activity (29.7% vs. 15.5%) (χ2 (1, *n* = 257) = 4.47; *p* = 0.035; Phi = 0.132).

### 3.2. Social Relationships

In the subscales related to social relationships, a MANOVA was performed in which the level of activity was considered as an independent variable and the dependent variables were the subscales that assessed the frequency and satisfaction related to social relationships; the analysis was statistically significant (*F* (4, 506) = 2.63, *p* = 0.034, *ƞ2* = 0.020). The group averages for the subscales are shown in Table 2.

In the ANOVAs testing continuation of social relationships depending on the level of activity, differences were found in the subscale of frequency of social relationships (Table 3) showing higher means for participants with a mild level of physical activity than those who perform a level of high activity (*p* = 0.011).

### 3.3. Functional Skills

On the functional skills scale, the results showed that the participants’ scores vary according to the level of physical activity they perform (*F* (2, 98.220) = 9.78, *p* < 0.001, *ƞ2* = 0.068), (Table 4) registering the differences between those who perform an intense level and a mild level of physical exercise (3.79 and 3.53 correspondingly) (*p* < 0.001).

Regarding the evaluation of the participants’ skill to fend for themselves (functional autonomy) (Table 5), the analyses showed that there are statistically significant differences in the autonomy that the individuals involved in the study have according to their intensity of physical activity (*χ2* (4, *n* = 257) = 18.77; *p* = 0.001) with a little connection between variables (VCramer = 0.191). The analysis to assess in what degrees of autonomy these differences in activity are indicates that they are registered between the degree of those who have regular autonomy with the other degrees (χ2 (2, *n* = 257) = 11.11; *p* = 0.004) with an association between the variables small (VCramer = 0.208) being the differences between those of intense activity with those of mild/low (1.9% vs. 11.8%) (*χ2* (1, *n* = 257) = 8.36; *p* = 0.004; *Phi* = −0.180) and between those of mild activity with those of intense/low (13.8% vs. 2.8%) (*χ2* (1, *n* = 257) = 10.65; *p* = 0.001; *Phi* = 0.204); and between the degree of autonomy with the other degrees (*χ2* (2, *n* = 257) = 14.68; *p* = 0.001) with a little connection between the variables (VCramer = 0.239) with the differences between those of intense activity with those of mild/low (78.9% vs. 56.9%) (*χ2* (1, *n* = 257) = 13.29; *p* < 0.001; *Phi* = 0.227) and between those of mild activity with those of intense/low (54.3% vs. 75.2%) (*χ2* (1, *n* = 257) = 12.31; *p* < 0.001; *Phi* = −0.219).

## 4. Discussion

The aim of this research was to evaluate the functional skills and autonomy, social relationships and subjective health of physically active older women based on the level of activity practiced. First, the results showed that both functional skills and autonomy were higher in women with a considerable degree of exercise. Regarding functional abilities, these were better in women with high levels of physical activity. To the contrary, higher degrees of autonomy were observed in women with mild physical activity. In any case, both functional skills and autonomy were higher in the more active women, with significant differences in autonomy between women with high and mild physical activity and those with low. These results could be explained by the benefits and protective effects that the routine of exercising can cause in the physical and cognitive state of the elderly [5,29,30,31,32]. In fact, with aging, subjects who remain active present lower losses in terms of physiological function, in addition to preserving muscle mass percentages to a greater extent [33]. In this line, a recent review after the analysis of 33 studies concluded that elderly persons with mild levels of physical exercise, which also included certain activities (physical, social, and cognitive) with a higher intensity, achieved a significant improvement in their physical capacity for the development of daily living activities [34]. These findings also support the theory that women who followed a strength-training plan obtained more significant enhancements in physical–functional ability, autonomy, and standard of living, compared to women who based their training solely on walking [35]. On the other hand, sedentary lifestyles seem to lead to the development of states of dependence for daily living activities in older adults [36].

Secondly, regarding social relationships, the results showed that women who engaged in mild physical activity in their daily routines tended to have social relationships more often. These results must be taken into consideration, since social relationships are an indicator of life expectancy, as well as an important factor for maintaining good physical and cognitive health [37,38]. In fact, loneliness is related to physical inactivity, functional impairment, increased depression, anxiety and stress [39,40]. For this reason, it is important to use strategies that can guarantee regular and quality social relationships, such as the practice of physical activity [41,42] as well as the support of spouses, family or friends [43].

Indeed, it was precisely the results of this study that indicated that the older women who showed the best subjective perception of health were those who had mild levels of physical activity, which in turn seem to be those who showed greater autonomy and frequency in social relationships. In this line, other authors emphasize the fact that maintaining high levels of inactivity or sedentary lifestyle entail a worse subjective perception of health [21], possibly due to the negative effects that these behaviours generate on the elderly, such as depression, anxiety, isolation, and functional and cognitive impairment, among others [44,45]. Recently, it was observed that better physical condition is related to better perception of health [25]. Therefore, it seems that maintaining an active lifestyle during aging could be related to a better perception of health and satisfaction about life [46] and improves functional skills, autonomy and ability to perform activities of daily living [47].

The present work contributes to provide scientific evidence through the experimental study of the benefits of physical activity practice and the encouragement of autonomy for activities of daily life so important to avoid dependency in a very representative population with a long-life expectancy, as is the case with women. It is highlighted that few works have paid attention to the study of physically active older women participating in sports or social centres that will represent the largest percentage of the population in the coming years. In the same way, few studies have assessed the high relevance of these variables for a total quality of life.

Such study will foster health promotion as well as specific interventions applied to older women, showing the previous scientific evidence from studies that indicate the benefits of the practice of physical activity.

As for practical implementations for society, these data represent evidence to promote engagement in physical practice in non-active women, given the benefits that are shown in this study, which among others are: avoiding functional dependence, increasing the social ties and socio-emotional benefits that this entails, and improving autonomy in women.

Regarding theoretical implications for the scientific community, this study contributes to the study of women and their health, as well as to the promotion, through evidence, of studies of physical practice in women so necessary in the sector of aging, health prevention, and women.

For future research, it would be interesting to carry out a longitudinal study in order to observe the changes that occurred such as improved strength, aerobic endurance, coordination, and balance in older women, as well as the socio-emotional benefits that have a very positive impact on women.

Nevertheless, one of the limitations of this study should be pointed out, and that is that it does not represent older people from other countries or the population of older women. However, what this study does show are the benefits in active women and those of the rest of the population that could be obtained through health promotion and intervention programs. On the other hand, the sample is made up of participants from 61 to 93 years old. This fact could influence the concern of importance that they give to maintain an active lifestyle as well as the degree of autonomy. Therefore, the results of this study should be interpreted with caution. For future research, it is recommended to divide the participants by age groups and perform an analysis taking into account the age factor. Finally, variables such as physical activity have been evaluated with the IPAQ questionnaire, a validated questionnaire widely used in different investigations; it is a subjective evaluation instrument. Therefore, it would be advisable to support this type of research with objective and precise instruments for measuring the level of physical activity, such as triaxial accelerometers.

## 5. Conclusions

To sum up, it could be argued that older women with intense levels of exercise have a higher standard of living in some of the personal aspects, obtaining better scores in functional skills being positively associated with their functional autonomy, as well as the increase in social relationships that are so relevant and enriching at this stage of life. Likewise, it is corroborated that the level of physical activity they develop is associated with their subjective perception of their health, with those with low activity levels registering more dissatisfaction with their health situation.

Overall, it seems that the practice of physical activity could help to maintain states of independence to carry out the activities of daily life; it also enhances social ties so important during aging, as well as the improvement of one’s own perception of health in benefit of the global improvement at the socio-emotional and physical level for the total quality of life of the elderly. With this, the presentation of this study aims to boost the fostering of promotion and health programs for older women in order to show the positive evidence that can be obtained and improve adherence through the establishment of programs for maintaining and improving the health of physically active women, as well as adherence to physical exercise practice in non-active women.

## Figures and Tables

**Table 1:** Frequencies of the subjective health subscale (CUBRECAVI) related to the level of physical activity.

	None		Some		Fairly		A Lot	
	*n*	*%*	*n*	*%*	*n*	*%*	*n*	*%*
Intense physical activity	4	3.8	20	19.2	69	66.4	11	10.6
Mild physical activity	11	9.5	32	27.6	50	43.1	23	19.8
Low physical activity	7	18.9	9	24.3	10	27.0	11	29.7

Note: *n* = number of participants; % = percentage.

**Table 2:** Means and standard deviations of the scores on the social relations subscales (CUBRECAVI) according to the level of activity.

	High P.A.		Mild P.A.		Low P.A.	
	*n*	*M (SD)*	*n*	*M (SD)*	*n*	*M (SD)*
Social rel. frequency	104	3.56 (3.76)	116	3.86 (0.72)	37	3.71 (0.77)
Social rel. Satisfaction	104	3.54 (0.54)	116	3.54 (0.50)	37	3.49 (0.45)

Note: *n* = number of participants; M = Mean; SD = Standard Deviation.

**Table 3:** ANOVAs testing continuation of social relationships depending on the level of activity.

	*F*	*Gl Effect*	*Gl Error*	*p*	*ƞ2*
Social relationships frequency	4.296	2	254	0.015	0.033
Social relationships satisfaction	0.151	2	254	0.860	0.001

Note: *p* < 0.025; F = statistic F; *p* = *p*-value; *ƞ2 =* eta square effect size.

**Table 4:** Means, Standard Deviation and One-way ANOVA of the participants’ scores on the functional skills scale (CUBRECAVI) according to their level of physical activity.

	Intense P.A.		Mild P.A.		Low P.A.		*F*	*p*	*ƞ2*
	*n*	*M (SD)*	*n*	*M (SD)*	*n*	*M (SD)*			
Functional skills	104	3.79 (0.39)	116	3.53 (0.49)	37	3.64 (0.47)	9.78	0.000 *	0.068

Note: *p* < 0.05. *n* = number of participants; M = Mean; SD = Standard Deviation F = statistic F; *p* = *p*-value. *ƞ2* = eta square effect size.

**Table 5:** Frequencies of the functional autonomy subscale (CUBRECAVI) according to their level of physical activity.

	Bad		Regular		Good		Very Good	
	*n*	*%*	*n*	*%*	*n*	*%*	*n*	*%*
High P.A.	0	0.0	2	1.9	20	19.2	82	78.9
Mild P.A.	0	0.0	16	13.8	37	31.9	63	54.3
Low P.A.	0	0.0	2	5.4	11	29.7	24	64.9

Note: *n* = number of participants; % = percentage.

## Data Availability

The database is under the custody of the IP with anonymized data.
